# Comment on Choi et al. Cellular Phone Use and Risk of Tumors: Systematic Review and Meta-Analysis. *Int. J. Environ. Res. Public Health* 2020, *17*, 8079

**DOI:** 10.3390/ijerph18105459

**Published:** 2021-05-20

**Authors:** Christopher Brzozek, Michael J. Abramson, Geza Benke, Ken Karipidis

**Affiliations:** 1 Australian Radiation Protection and Nuclear Safety Agency, Melbourne, VIC 3004, Australia; ken.karipidis@arpansa.gov.au; 2 School of Public Health and Preventive Medicine, Monash University, Melbourne, VIC 3004, Australia; michael.abramson@monash.edu (M.J.A.); geza.benke@monash.edu (G.B.)

In September 2020, Choi et al. published their paper “Cellular Phone Use and Risk of Tumors: Systematic Review and Meta-Analysis” in the *International Journal of Environmental Research and Public Health* [1]. This systematic review and meta-analysis concluded that mobile (cellular) phone use was associated with increased tumor risk despite the main meta-analysis finding no association. Choi et al. analyzed 3 sub-groups of studies (Hardell, INTERPHONE, and other studies), which found an increased risk in the Hardell subset (OR: 1.15; 95% CI: 1.00, 1.33), a decreased risk in the INTERPHONE subset (OR: 0.81; 95% CI: 0.75, 0.88), and no significant association in the others (OR: 1.02; 95% CI: 0.92, 1.13) [1]. However, there are important methodological issues and incorrect interpretations in the review by Choi et al. that need to be clarified.

Firstly, the INTERPHONE group were unfairly and repeatedly criticized for being funded by the cellular phone industry, even whilst acknowledging agreements that guaranteed the study’s complete scientific independence. The claim that many investigators of the INTERPHONE study were reliant upon industry funding and therefore may have “hidden conflicts” of interest was conjecture and supported only by a Hardell reference [2].

Secondly, the authors argued that the Hardell subset of studies were of higher quality compared to the INTERPHONE studies. Although the Hardell studies were similar to the INTERPHONE studies, there were subtle methodological differences in recruitment, subject age and status, exclusion criteria, data collection, definition of regular phone use etc. which could account for the different results. A closer look at the methodological differences does not show the Hardell studies to be of higher quality than the INTERPHONE studies. Furthermore, the Hardell group showed methodological variation within their own studies [3]. In contrast, the INTERPHONE results came from 8 independent research groups, which all followed a common protocol. Therefore, it is difficult to support the authors’ conclusion that the Hardell studies were of a higher quality.

The Hardell studies included a wider age range (generally 20–80 years) compared to the INTERPHONE studies (generally 20–69 years). Although a greater age-range increased the sample size of the study, it did not materially add to power and may lead to the inclusion of tumors with recognized different etiology [4]. The choice of age-range by INTERPHONE aimed to maximize the likelihood of exposure [5]. Additionally, the Hardell studies reported participation rates of 82–91% in cases and 80–92% in controls (apart from Hardell et al., 2010) [6]. Nevertheless, if the definition of eligible cases used the exclusion criteria of the INTERPHONE studies, then the participation rate of the Hardell studies would have been between 65–85% [7].

Exposure misclassification remains a prominent issue in both groups of studies with Hardell defining “any use” as regular phone use. This is questionable because it includes casual phone users. If mobile phones truly cause cancer, but only at higher exposures, employing such a generous definition of regular use means that the effect might be diluted. INTERPHONE defined regular use as once per week on average during at least 6 months (it could be argued that even the INTERPHONE definition of regular use was too generous).

Finally, the meta-analysis which pooled different types of case-control studies and tumor types together is limited, as these tumors have different etiologies and no viable biological mechanism to how a cellular phone use exposure could cause these various tumors [8]. The authors claim this as a strength, but it is in reality a limitation since it is unlikely cell phones cause cancer in the various cell types of all the body sites included in the meta-analysis. Cellular phone use emits non-ionizing (radiofrequency) radiation which, unlike ionizing radiation, lacks the ability to cause molecular damage in all types of tissue. Consequently, it is difficult to draw any meaningful conclusions from this approach. Based on the detailed methodological issues and uncertain interpretations, it is our opinion that this systematic review and meta-analysis does not present substantial evidence of a link between cellular phone use and tumor risk.
